# News and Notes

**Published:** 1901-06

**Authors:** 


### NEWS AND NOTES.

The *Atlanta Medical and Surgical Journal* and *Southern Medical Record* having been consolidated into the Atlanta Journal-Record of Medicine, exchanges will please be sent only to the latter. We are getting many duplicates.

The post-office authorities of Washington, says *American Medicine*, are now investigating the case of a doctor who advertised to cure deafness, without fail, for $18.50. Persons sending this amount were forwarded 2,000 pills, with instructions to take one each day, and on no account to miss a day or the charm would be broken, and it would be necessary to start all over again. As the truth of the doctor's claim cannot be proved until the end of five and a half years, the authorities are puzzled what course to take.

A surgeon's sewing machine, says *American Medicine*, was exhibited by Dr. Paul Michel at the late Congress of Medicine. The instrument is quite small, easily held in the hands, and has received the Barbier prize of the Faculté de Médicin. In future a surgeon need not slowly stitch the edges of a wound. With the left hand he keeps the two lips together, and with the right he fastens it by means of little clasps or "agrafes" of nickel having points which only penetrate the epidermis, and are not painful. These catches are applied to the machine, a species of pincer armed with them, which can be disinfected by heating it red-hot.

**This Journal and Atlanta Semi-weekly Journal one year for $1.00 cash in advance. No checks. Strictly to new subscribers.**

THE *Philadelphia Medical Journal* quotes the story of a remarkable surgical operation told in a Danish medical periodical relative to the treatment of a patient who had become asphyxiated from the administration of chloroform. The operating surgeon was a certain Dr. Maag, but the method which he had employed had previously been suggested by Dr. Prus, of Lemberg. A laborer, twenty-seven years old, who had suffered from sciatica, was to be operated upon to relieve that trouble. Chloroform was given, and the operation begun. The patient struggled, however, and when the process of anesthesia was carried further he stopped breathing. Several expedients were resorted to in order to restore respiration, but in vain. There was no longer any pulse. In this emergency Dr. Maag opened the chest, detached portions of the third and fourth ribs two and a half inches long, and turned them back with a flap of flesh. Through the opening thus made he thrust his hand. The heart was firmly grasped and compressed rhythmically. After a few squeezes that organ began to beat naturally. It was necessary to employ compression again at times, and also to inflate the lungs artificially. But by these means the patient was kept alive for eleven and a half hours, and Dr. Maag is inclined to believe that the man would have recovered were it not that one of the pleuræ was accidentally punctured.

